# Supplementary material for: Identification of Immune Cell Components in Breast Tissues by a Multiparametric Flow Cytometry Approach
Source: Cancers (Basel). 2022 Aug 10;14(16):3869. doi: 10.3390/cancers14163869 (PMC9406207; doi:10.3390/cancers14163869)
Supplement: Supplementary file 1 [file cancers-14-03869-s001.zip › cancers-1819479-supplementary.pdf]

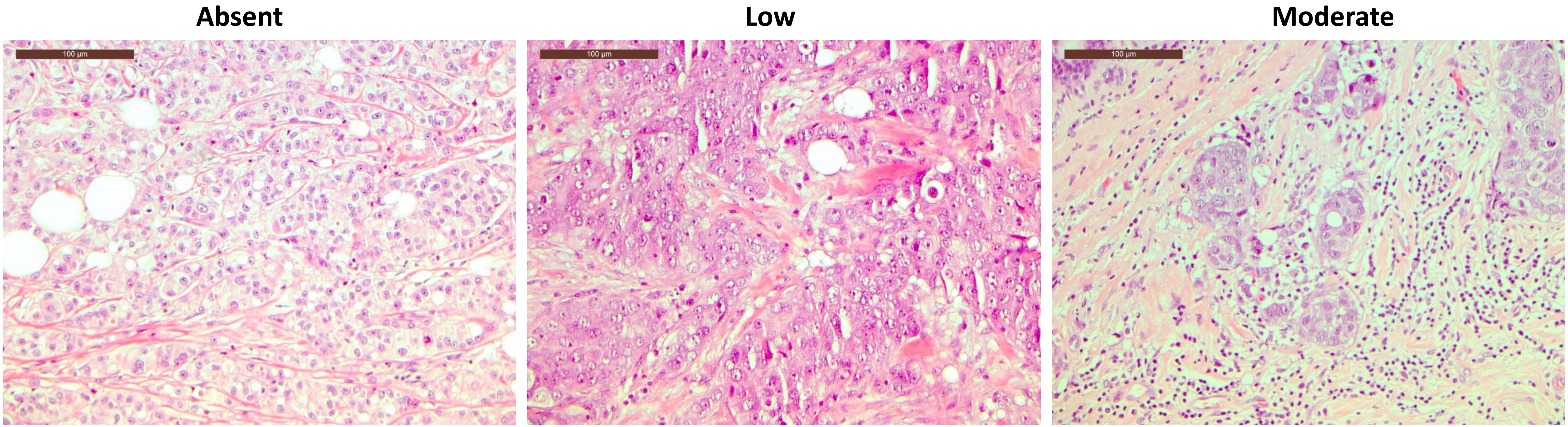

Supplementary Figure S1. The hematoxylin-eosin staining of three different breast cancer tissue patients with three different level of TILs, Absent (left panel), Low (middle panel) and moderate (right panel). The inset represent an enlarged detail of TILs. Magnification 20×. Scale bars 100 µm.

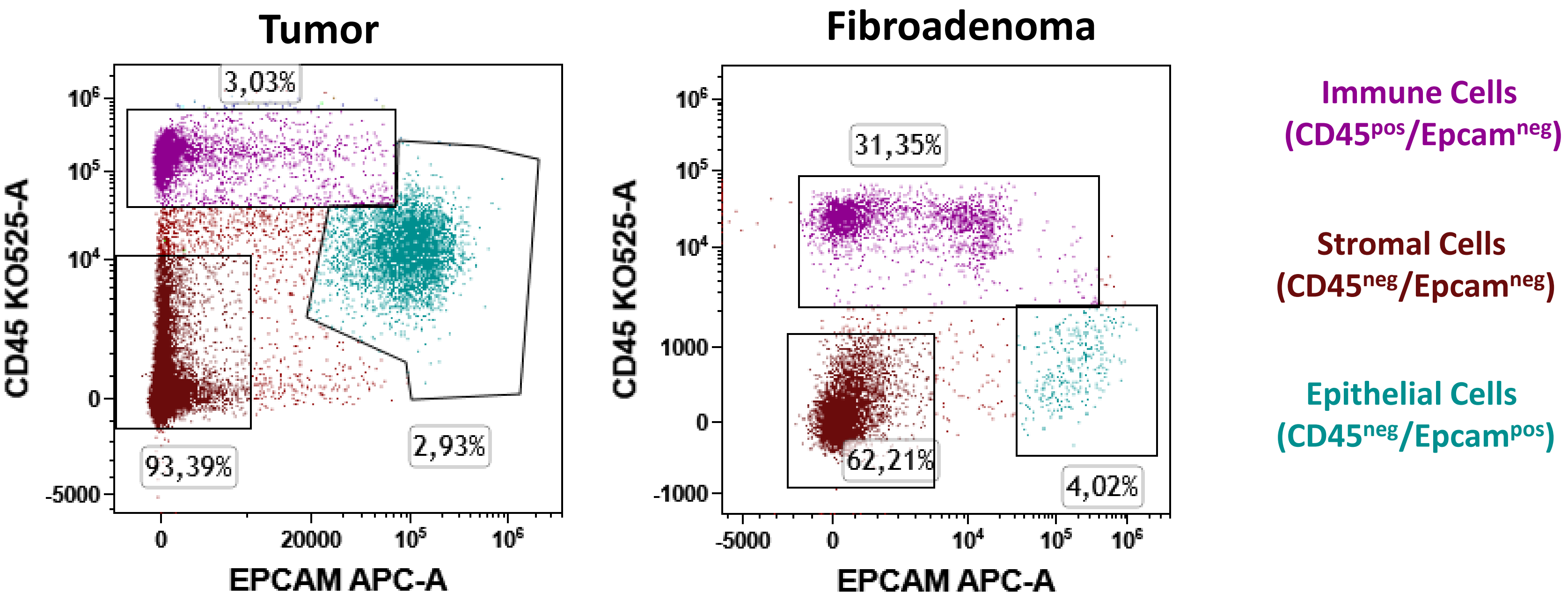

Supplementary Figure S2. Representative CD45vsEPCAM dot plot of Tumor (A) and Fibroadenoma (B) recovered cells. The dot plot analisys shows the selection of Immune cells (purple dots, CD45^{pos}/Epcam^{neg}), Stomal Cells (grenade dots, CD45^{neg}/Epcam^{neg}) and Epithelial Cells (light blue dots, CD45^{neg}/Epcam^{pos}). Numbers represent the percentage of gated cells.

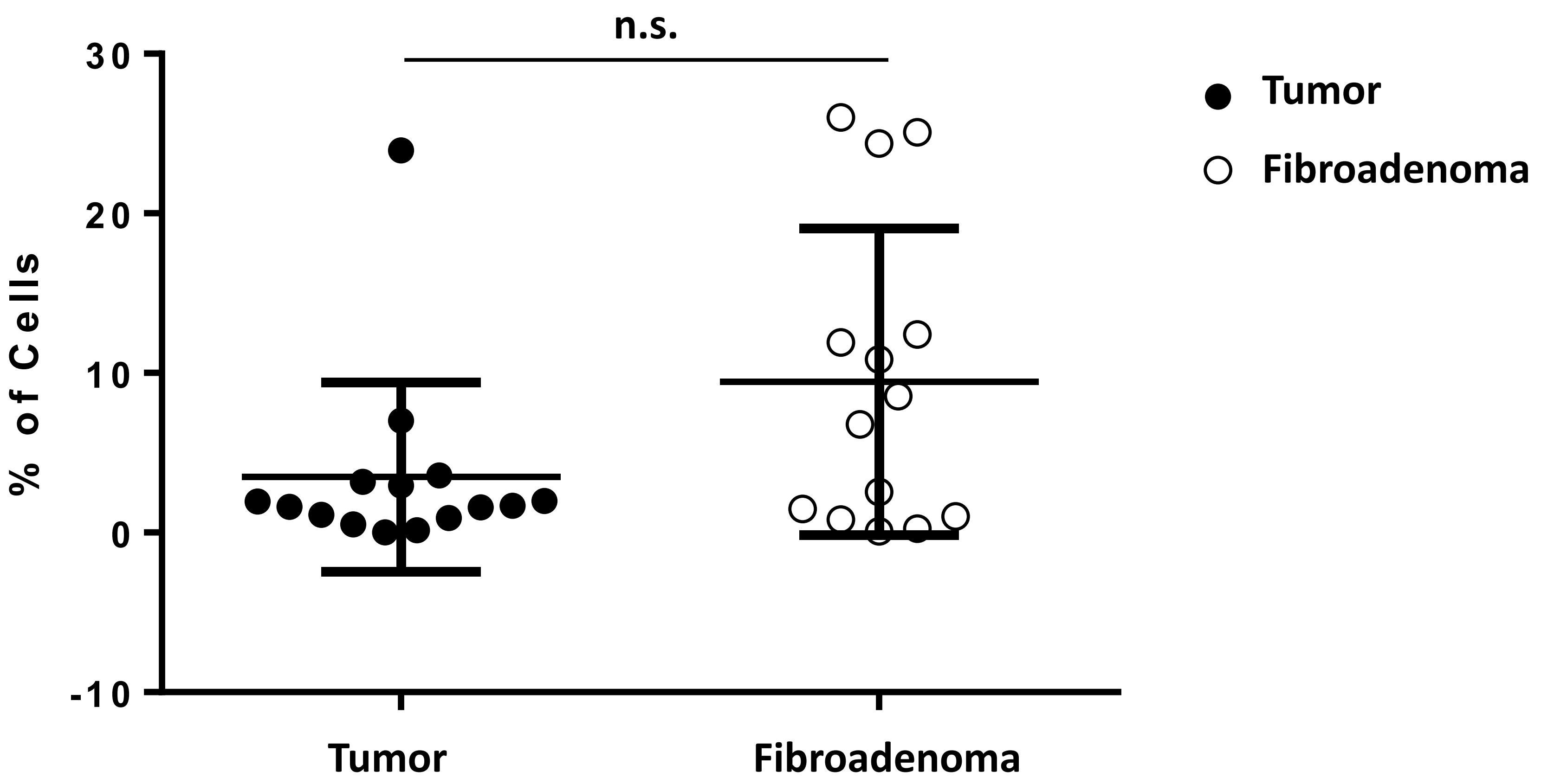

Supplementary Figure S3. B-Lymphocytes were plotted, according to specific membrane receptor, in Tumor (black dots) and Fibroadenomas (white dots) tissue patients. n.s. = Not significative
